# Therapeutic Application of an Ag-Nanoparticle-PNIPAAm-Modified Eggshell Membrane Construct for Dermal Regeneration and Reconstruction

**DOI:** 10.3390/pharmaceutics14102162

**Published:** 2022-10-11

**Authors:** Emily Briggs, Rosemond A. Mensah, Kapil D. Patel, Nandin-Erdene Mandakhbayar, Nik San Sharifulden, Zalike Keskin Erdogan, Lady V. Barrios Silva, Kawther Salim, Hae-Won Kim, Linh T. B. Nguyen, David Y. S. Chau

**Affiliations:** 1 Eastman Dental Institute, University College London, Royal Free Hospital, Rowland Hill Street, London NW3 2PF, UK; 2 Department of Materials, Henry Royce Institute, The University of Manchester, Rumford Street, Manchester M13 9PL, UK; 3 School of Cellular and Molecular Medicine, University of Bristol, Queens Road, Bristol BS8 1QU, UK; 4 Institute of Tissue Regeneration Engineering, Dankook University, Cheonan 31116, Korea; 5 UCL Eastman-Korea Dental Medicine Innovation Centre, Dankook University, Cheonan 31116, Korea; 6 Department of Nanobiomedical Science, Dankook University, Cheonan 31116, Korea; 7 BK21 NBM Global Research Centre for Regenerative Medicine, Dankook University, Cheonan 31116, Korea

**Keywords:** drug-delivery, biomaterial, crosslinking, thermoresponsive, transglutaminase, wound dressing, eggshell membrane

## Abstract

Current therapeutic treatments for the repair and/or replacement of damaged skin following disease or traumatic injury is severely limited. The chicken eggshell membrane (ESM) is a unique material: its innate physical and mechanical characteristics offer optimal barrier properties and, as a naturally derived extract, it demonstrates inherent biocompatibility/biodegradability. To further enhance its therapeutic and clinical potential, the ESM can be modified with the thermo-responsive polymer, poly(N-isopropylacrylAmide) (PNIPAAm) as well as the incorporation of (drug-loaded) silver nanoparticles (AgNP); essentially, by a simple change in temperature, the release and delivery of the NP can be targeted and controlled. In this study, ESM samples were isolated using a decellularization protocol, and the physical and mechanical characteristics were profiled using SEM, FT-IR, DSC and DMA. PNIPAAm was successfully grafted to the ESM via amidation reactions and confirmed using FT-IR, which demonstrated the distinctive peaks associated with Amide A (3275 cm^−1^), Amide B (2970 cm^−1^), Amide I (1630 cm^−1^), Amide II (1535 cm^−1^), CH_2_, CH_3_ groups, and Amide III (1250 cm^−1^) peaks. Confirmation of the incorporation of AgNP onto the stratified membrane was confirmed visually with SEM, qualitatively using FT-IR and also via changes in absorbance at 380 nm using UV-Vis spectrophotometry during a controlled release study for 72 h. The biocompatibility and cytotoxicity of the novel constructs were assessed using human dermal fibroblast (HDFa) and mouse dermal fibroblast (L929) cells and standard cell culture assays. Metabolic activity assessment (i.e., MTS assay), LDH-release profiles and Live/Dead staining demonstrated good attachment and spreading to the samples, and high cell viability following 3 days of culture. Interestingly, longer-term viability (>5 days), the ESM-PNIPAAm and ESM-PNIPAAm (AgNP) samples showed a greater and sustained cell viability profile. In summary, the modified and enhanced ESM constructs were successfully prepared and characterized in terms of their physical and mechanical profiles. AgNP were successfully loaded into the construct and demonstrated a desirable release profile dependent on temperature modulation. Fibroblasts cultured on the extracted ESM samples and ESM-PNIPAAm demonstrated high biocompatibility in terms of high cell attachment, spreading, viability and proliferation rates. As such, this work summarizes the development of an enhanced ESM-based construct which may be exploited as a clinical/therapeutic wound dressing as well as a possible application as a novel biomaterial scaffold for drug development.

## 1. Introduction

Damage to the skin can result from several insults, including burns, traumatic injury, cancer, and infection. Physiologically, wound healing is a multi-parameter and complex process which is dependent on the synchronised activation of several cell types and cytokine mediators [1]. Furthermore, pathologies such as diabetes mellitus or peripheral vascular disease can result in chronic wounds, which can further develop and/or increase the potential of infection and additional complications [2,3]. During aberrant tissue repair, impaired angiogenesis and nutrient delivery result in persistent infection and the continuous activation of inflammatory cells in the wound bed. As such, patients suffer from non-healing open wounds (ulcers), which require continuous clinical management. Annually, the cost of managing such wounds is estimated to be £5.3 billion in the UK [4] and further studies have reported that the frequency of such wounds to be increasing at a rate of 12% per year, highlighting the critical requirement for strategies to improve healing rates in chronic dermal wounds [5].

Traditional dressings, made of cotton gauze, fail to provide a moist environment and often become adherent to the wound bed [6]. Modern dressings have been developed to improve healing by facilitating regeneration by providing a permeable and moist environment. A range of materials have been explored, including natural and synthetic hydrocolloids [7], semi-permeable hydro films [8], and drug-loaded therapeutic dressings [9,10].

**Figure 1:**
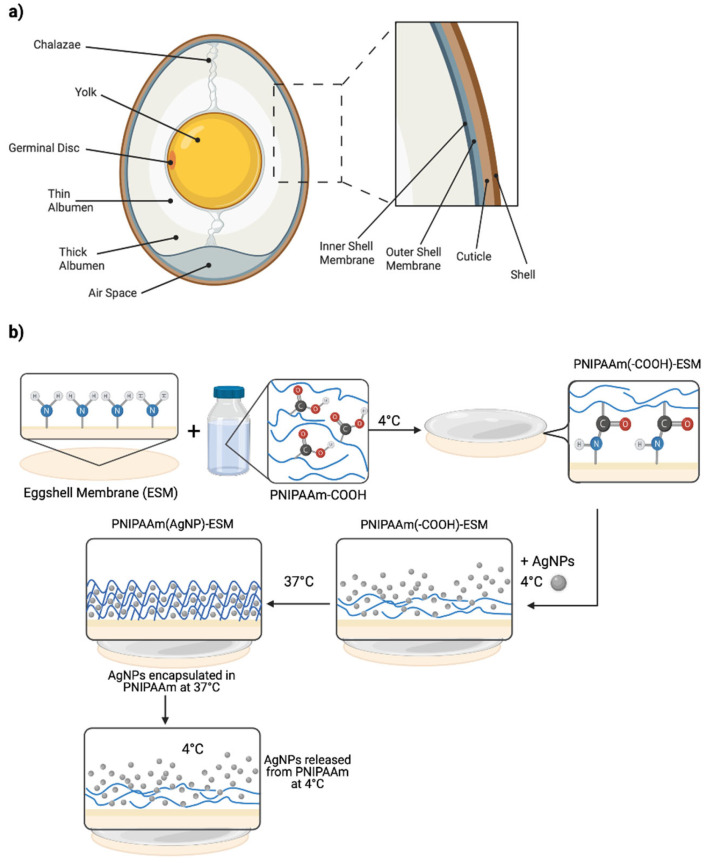
Schematic outing the fabrication of the ESM-PNIPAAm (AgNP). (**a**) Diagram of chicken (gallus gallus) eggshell membrane. Inset shows the image of Raw ESM extracted with acetic acid (0.5 M) at room temperature (19 °C). (**b**) PNIPAAm is grafted to the surface of the ESM via the amidation reaction between the NH2-rich ESM and the COOH-terminated PNIPAAm hydrogel. The AgNPs are “loaded” onto the conjugated membranes at 4 °C (<LCST of PNIPAAm). Upon incubation at 37 °C, the hydrogel undergoes phase transition, and the NP are encapsulated within the crosslinked gel. In principle, when the temperature is lowered (<LCST), the hydrogel becomes hydrophilic, and the NP are release into the environment.

The eggshell membrane (ESM) is a highly collagenous, thin, fibrous membrane found sandwiched between the calcified shell and albumin of chicken eggs (Figure 1a). Consisting of an inner and an outer layer, the membranes are arranged to form a semi-permeable barrier [11]. Recent studies suggest the material’s physical properties and inherent biocompatibility make it an ideal candidate to enhance tissue regeneration in corneal/ophthalmologic application [12]. In a preliminary study, Yang et al. report the direct application of the ESM to the site of skin grafts enhanced pain management and wound protection [13]. In addition, both Jun et al. [14] and Guarderas et al. [15] reported (native) ESM accelerates wound closure and regeneration in the early stages of wound healing. Interestingly, as ESM is composed of collagenous material, it may be possible to further enhance the mechanical properties using traditional crosslinking methods, e.g., glutaraldehyde, NHS-EDC, transglutaminase and physical techniques [16], and is currently an area of research interest.

In contrast, the membrane can additionally be modified to further enhance its therapeutic potential. Herein, we report the modification of the ESM with the stimuli-responsive hydrogel polymer poly(*N*-isopropylacrylAmide) (PNIPAAm), alongside the incorporation of drug-loaded nanoparticles (NP). PNIPAAm is a widely studied thermoresponsive hydrogel: the key attribute being its lower critical solution temperature (LCST) of ~32 °C [17] which, accordingly, causes the polymer to undergo a phase transition to a hydrophobic globule conformation at temperatures above its LCST. As such, surfaces coated with PNIPAAm can therefore alternate between a hydrophilic and a hydrophobic state depending upon changes to the external temperature, causing adherent agents, such as antibacterial silver NP (AgNP) [18], to become detached and consequently released into the environment [19,20]. However, despite being studied as a vehicle for drug delivery systems, applications that demonstrate the use of PNIPAAm-based hydrogels systems in wound healing are limited.

Although widely available as food waste, few articles report the complete characterisation of the ESM as a potential material for biomedical applications. Furthermore, to date, no studies have proposed the application of the ESM grafted with an AgNP-loaded PNIPAAm hydrogel. The work herein summarises the isolation and characterisation of a variety of modified ESM samples in terms of their physical, chemical, biological and drug delivery profiles in the context of potential therapeutic applications to (dermal) wound healing.

## 2. Materials and Methods

Large, free-range, brown chicken eggs (British Blacktail, *Gallus gallus* domesticus) were purchased from a local supermarket (Sainsbury’s, London, UK). Acetic acid (AA) (glacial, 99.7%) was purchased from Alfa Aesar (36289, Heysham, UK). Sodium hydroxide (NaOH, 98%) (71687), N-(3-Dimethylaminopropyl)-N’-ethylcarbodiimide hydrochloride (EDC, crystalline) (E6383), N-Hydroxysuccinimide (NHS, 98%) (130672), Lugol’s solution (Potassium iodide solution) (32922), Poly(N-isopropylacrylAmide) (724459), penicillin/streptomycin (P/S) (P4333), MES hydrate (99.5%) (M2933) and silver dispersion solution (0.02 mg/mL in aqueous buffer) (730785) were purchased from Sigma-Aldrich (Poole, UK). Dulbecco’s modified Eagle medium (DMEM) (21331046), foetal bovine serum (FBS) (A4766801), AlamarBlueTM (DAL1025), paraformaldehyde (PFA, 4%), Live/DeadTM cytotoxicity kit (L3224), phosphate-buffered saline (PBS, pH 7.2) (20012019) and Human Dermal Fibroblast (HDFa) (C0125C) cells were purchased from GIBCO (Thermo Fisher, Paisley, UK). CellTiter 96^®®^ AQueous One Solution Cell Proliferation assay (i.e., MTS assay) (G3582) and the CytoTox 96^®®^ Non-radioactive Cytotoxicity Assay (i.e., LDH assay) (G1780) were purchased from Promega (Southampton, UK).

### 2.1. Membrane Isolation

The ESM was derived from large chicken eggs; the eggshell was removed using an optimised decellularization protocol, as previously reported [12]. Eggs were submerged in acetic acid (AA, 0.5 M) at room temperature (~19 °C) for 44 h before being removed from the acid and rinsed in distilled water. Remaining residual shell (calcium carbonate) was manually removed. The albumin and yolk were removed, and the remaining membrane carefully rinsed. Membranes were refrigerated (4 °C) in distilled water in order to avoid dehydration.

### 2.2. Membrane Modification

ESM-PNIPAAm (AgNP) Fabrication. The ESM-PNIPAAm was fabricated as previously reported by Nguyen et al. [21], outlined in Figure 1b. An activation solution was prepared by adding MES hydrate (0.195 g, 0.05 M), NHS (0.138 g, 0.06 M) and EDC (0.46 g, 0.12 M) to 20 mL of deionised water. The pH of the solution (Solution A) was confirmed to be 6 using pH indicator strips (PANPEHA). Carboxyl-terminated PNIPAAm-COOH (2 g, 10 *w*/*v*) was mixed into 20 mL of Solution A for 3 h on a magnetic stirrer to activate the solution (Solution B). The ESM was cut into discs using an AcuPunch^®®^ (5 mm), submerged in 20 mL of solution B in a petri dish, and continuously stirred (50 rpm) at 4 °C for 24 h. ESM-PNIPAAm discs were removed from solution B and inserted into a 24-well plate with 200 μL of distilled water to avoid dehydration. To load the AgNP, the water was removed, and 100 μL of silver dispersion was added dropwise in each well using a micropipette, covered with foil, then shaken at 4 °C for 24 h. Modified samples were then dried for 3 h in a fume hood and stored at 4 °C. The grafting of the ESM-PNIPAAm was confirmed using Fourier transform infrared red (FT-IR) spectroscopy as well as scanning electronic microscopy (SEM) analysis.

Enzymatic Crosslinking. ESM samples were biologically crosslinked using the enzyme transglutaminase (TG). Stock transglutaminase solution was prepared by dissolving 30 mg of TG powder “Meat Glue” (Special Ingredients Ltd., Chesterfield, UK) to 30 mL of PBS. Raw ESM samples were submerged in the solution and incubated (37 °C) for 24 h. The crosslinked membranes (TG-ESM) were then rinsed before being stored at 4 °C in PBS.

### 2.3. Material Characterisation

Morphological Analysis. A scanning electron microscope (SEM) (Zeiss EVO HD, Jena, Germany) was used to image and analyse the inner and outer surfaces of the ESM with and without AgNP. Before examination, specimens were cut into 12 mm discs and fixed in PFA (200 µL, 4%) for 48 h. Samples were then coated (95% gold and 5% palladium, Polaron E5000 Sputter Coater, Quorum Technologies, Laughton, UK). 500× and 5000× magnifications were used to visualise the surface morphology of the samples.

Biochemical Analysis. Fourier transform infrared spectrophotometer (FT-IR) (Spectrum One, Perkin Elmer, Llanstrisant, UK) was used to characterise the biochemical composition of the ESM samples, and to confirm the fabrication of the ESM-PNIPAAm. Samples were scanned in the within the 400 to 4000 cm^−1^ range, at room temperature (19 °C), and calibrated by taking the initial background absorbance. Time Base (Spectrum) software was used to process the spectra.

Mechanical Analysis. Tensile strength tests were performed on saturated rectangular samples (~10 × 17 mm, pre-soaked overnight in PBS) at room temperature (19 °C) using tensile film clamp as part of the Dynamic Mechanical Analysis (DMA850, TA Instruments, New Castle, NSW, USA) setup. The TRIOS software was used to determine both the ultimate tensile strength (UTS), and % elongation at break. *Young’s Modulus* was extrapolated from the linear slope of the stress-strain curves as stated below:

$$\mathrm{Young}’s\mathrm{Modulus}\;(MPa)=\frac{S\;\left(MPa\right)\;}{e\;\left(\%\right)}\;$$

[where *S* = change in stress, *e* = change in strain].

Thermal Analysis. Differential scanning calorimetry (DCS25, TA Instruments, New Castle, NSW, USA) was used to track variations in the heat capacity of membrane samples. Weighed samples were inserted into Tzero^®®^ Pans and Lids. A control empty pan was used as a reference and runs were performed at least in triplicate. Pans were heated from 0 °C to 250 °C, at a ramp rate of 20 °C/min, under a continuous flowrate of nitrogen gas. TRIOS software was used to analyse and report the data.

Contact Angle Measurements. The response of the raw and modified ESM samples to water was determined by measuring the contact angles (CA) using the sessile drop method/optical contact angle profiling. In brief, a droplet of distilled water (~2 µL) was deposited on the sample surface and the CA measured at room temperature (~19 °C), using a CAM 200 optical angle meter (KSV Instruments Ltd., Helsinki, Finland). 

Drug Release Profile of ESM-PNIPAAm (AgNP). The drug release profile and thermoresponsive properties of the ESM-PNIPAAm (AgNP) was analysed by measuring the absorbance of AgNP within PBS solutions containing the modified membranes, at 380 nm. In total, 12 mm (disc diameter) samples were immersed in 1.5 mL PBS, at either 4 °C or 37 °C, to demonstrate the effects of temperatures above and below the LCST. The solutions were removed at hourly intervals, for a total of 6 h, to measure the ‘initial burst’ release profile, and then at 24, 48, and 72 h to determine the ‘absolute’ release profile. Samples were decanted into cuvettes (accurate for minimum 285 nm wavelength and over) and analysed using an UV-Visible spectra (UNICAM UV 500 Spectrophotometer, Spectronic, London, UK). The concentration of NP released was quantified against a calibration curve of known concentrations. Vision 1 was used to analyse the data.

### 2.4. Biological Characterisation

Cytotoxicity analyses. Human dermal fibroblasts (adult) (HDFa) were cultured in T-75 flasks (Corning Life Sciences, UK) with 13 mL of DMEM (Life Technologies Ltd., Paisley, UK) supplemented with 10% FBS, 2 mM L-glutamine (LG), and 100 U/mL penicillin, and 100 μg/mL streptomycin (P/S) under standard humidified cell culture conditions (37° C and 5% CO_2_). Routine cell culture involved a standard trypsinisation protocol (i.e., 1% (*v*/*v*) trypsin-EDTA) every 3 days including gentle rinsing with PBS (~10 mL). Cells at passage 8 (P8) were used in subsequent experiments. For Live/Dead staining, L929 mouse fibroblast cells were employed. L929 cells were cultured as described above.

Sample membranes were cut into 5 mm (diameter) discs before being placed into 96-well plates (Corning Costar^TM^, Thermo Fisher, Paisley, UK) and UV sterilised (Steristorm 2537a) for 20 min. Thereafter, samples were soaked, in PBS, for 1 min for equilibration. HDFa cells were seeded on the inner side of the membranes (Raw ESM, ESM-PNIPAAm, ESM-PNIPAAm (AgNP), I-ESM and TG-ESM) at a density of 1 × 10^2^ cells/mL in 150 µL of the complete growth media and incubated accordingly (37 °C and 5% CO_2_). Each sample group had 6 replicates and groups of tissue culture plastic (TCP) were included in each plate as a control.

The metabolic activity of the cells was evaluated using the CellTiter^®®^ 96 Aqueous One Solution Cell Proliferation assay (Promega, Southampton, UK) according to the manufacturer’s protocol. In summary, following 1, 3, 5, 7, 10, and 14-days incubation, 50 µL of the culture media was removed from each well before being transferred into a new 96-well plate and retained for the LDH assay. For the proliferation assay, 20 µL of CellTiter One reagent was added to each well and incubated at 37 °C for 90 min whilst wrapped in aluminium foil. Following incubation, the supernatant solution was transferred to a new plate and read at 490 nm using a Tecan Infinite M200 microplate reader (Tecan, Switzerland).

Lactate dehydrogenase (LDH) release from the cells was quantified using the CytoTox 96^®®^ Non-radioactive Cytotoxicity Assay kit (Promega, Southampton, UK). In total, 50 µL of Reagent A was added to 50 µL of media suspension in each well (transferred to new plate as previously described), which was then incubated and covered in aluminium foil at ~19 °C for 30 min. Thereafter, 25 µL of stop solution was added to each well. The absorbance was the immediately read using a Tecan Infinite M200 microplate reader (Tecan, Männedorf, Switzerland). 

To support the quantitative assays, Live/Dead^TM^ staining was used to demonstrate the biocompatibility of the raw and modified membrane samples (ESM-PNIPAAm and ESM-PNIPAAm (AgNP)). A total of 100 µL of L929 (P11) cells, at a density of 1 × 10^4^ cells/mL, were seeded onto membrane samples (12 mm discs) in 24-well plates. At the relevant time point, the media was discarded, and the samples were rinsed with PBS. The stain was prepared by adding 20 µL of EthD-1 (2 mM) stock solution to 10 mL PBS, combined with 5 mL Calcein AM (4 mM) stock solution. After 3 and 5-days incubation, 100 µL of the stain was added to each sample and incubated (~19 °C) for 20 min. The viability of the cells was observed using fluorescence microscopy (LEICA Instruments, Milton Keynes, UK) on Image Capture Pro software.

### 2.5. Statistical Analysis

Quantitative results are expressed as mean and standard deviation (SD). Experiments were performed in triplicate unless otherwise stated. Significance of each dataset was determined using a student’s *t*-test with the statistical significance indicated by (*), which corresponds to a *p* < 0.05, (**) which corresponds to a *p* < 0.01 and (***), which corresponds to a *p* < 0.001. For the physical characterisation of membrane, modified samples were compared to the raw ESM independently. The data were analysed using Microsoft Excel 2019 and presented using Origin 2020. Microscopy images were developed using ImageJ software.

## 3. Results and Discussion

### 3.1. Material Characterisation

**Figure 2:**
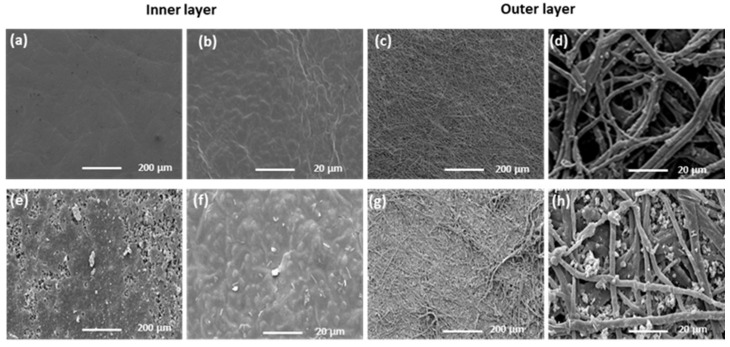
SEM images of the extracted ESM samples. (**a**,**b**) Inner layer of the membrane at magnification of 500× and 5000× respectively. (**c**,**d**) Inner layer of the membrane at magnification of 500×, and 5000× respectively. (**e**,**f**) inner layer of ESM-PNIPAAm (AgNP) at magnification of 500×, and 5000× respectively. (**g**,**h**) outer layer of ESM-PNIPAAm (AgNP) at magnification of 500×, and 5000×, respectively.

Morphology. Following extraction via acid decellularization, the raw ESM was analysed using SEM; images are shown in Figure 2. The images demonstrate that distinct structural patterns between the inner and outer surface of the membrane exist. The inner surface shows a flat, continuous morphology in which multiple circular bulges are visible upon higher magnification (Figure 2a,b). In contrast, the outer surface displays a dense unorganised fibrillar network containing microporous structures. (Figure 2c,d). The diameter of these fibres was measured as 1.778 ± 0.778 μm. Understanding the structure of the egg, the structural properties of the two surfaces correspond to their position on the eggshell. For example, the fibrils in the outer layer pay a critical role in early development, as nucleation sites on the membrane attract calcium salts, forming the eggshell [22]. The deposition of the AgNP on both the inner and outer layers of the ESM are observed in Figure 2e–h.

**Figure 3:**
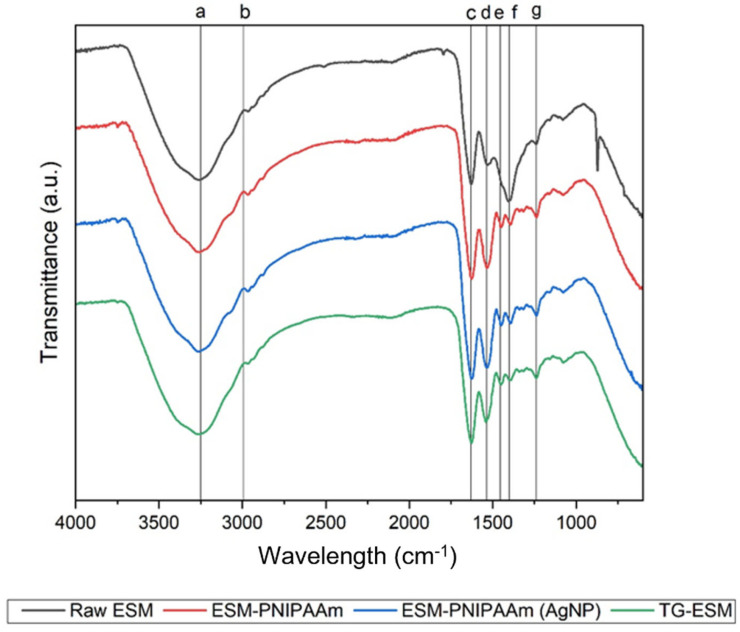
Fourier transform infrared spectrophotometer (FT-IR) spectra. Representative FT-IR summarising the chemical bonding structure in the raw ESM, as well as alterations results from modification protocols, i.e., raw ESM and modified ESM (ESM-PNIPAAm, ESM-PNIPAAm (AgNP), TG-ESM). Vertical lines (a, b, c, d, e and f) identify the distinctive peaks associated with the bands: Amide A (3275 cm^−1^), AAmide B (2970 cm^−1^), AAmide I (1630 cm^−1^), AAmide II (1535 cm^−1^), CH_2_, CH_3_ groups, and Amide III (1250 cm^−1^) respectively. Unlike the raw ESM, spectra of the modified membranes show a shift of the AAmide II peak (1535 cm^−1^) at a higher intensity and additional smaller peaks within the wavelength region of 1300–1450 cm^−1^, indicating CH_2_ and CH_3_ groups and C-H bending.

Biochemical Properties. Chemical bonding in the raw ESM samples were observed using FT-IR within the 400–4000 cm^−1^ range. Referring to Figure 3, the spectra summarise the functional groups within the raw samples within which Amide A and B bands can be identified. The Amide A band (Figure 3a) characterised as the broad peak at 3275 cm^−1^ corresponds to hydrogen bonds and C-H stretching. The smaller peak observable at 2910 cm^−1^ is characteristic of typical Amide B bands (Figure 3b), corresponding to the C-H bonds found in the =C-H functional group. Similar spectra and biochemical characteristics have previously been reported in the literature [12,23,24,25,26]. Amide band I (Figure 3c), Amide band II (Figure 3d) and Amide band III (Figure 3g), visualised as peaks 1630 cm^−1^, 1535 cm^−1^, 1250 cm^−1^ respectively, are characteristic of the membrane’s high protein content, specifically collagen. The Amide I and II bands represent major bands of the protein infrared spectrum and can be associated with C=O stretching, N-H bending and C-N stretching vibrations [23].

Additionally, FT-IR was implemented to characterise the modified membranes (ESM-PNIPAAm, ESM-PNIPAAm (AgNP), ESM-I and TG-ESM) (Figure 3). The technique confirmed the successful grating of PNIPAAm to the ESM, evident by a small shift and increased Amide II peak (1535 cm^−1^) intensity. This indicates conjugation of C=O, C-N and increased N-H stretching within the modified membrane [27]. This is further supported by the presence of CH_3_ (Figure 3e) and CH_2_ (Figure 3f) peaks visible between 1300–1450 cm^−1^ on the spectra of the grafted membrane not visible on that of the raw ESM. A similar spectrum can be observed for the TG-ESM and I-ESM in which CH_3_ and CH_2_ peaks can be observed. Interestingly, the FT-IR profile of the modified membrane (ESM-PNIPAAm (AgNP)) has a profile matching that of ESM-PNIPAAm, suggesting that “loading” AgNP to the gel does not further alter the chemical composition.

### 3.2. Thermal Properties

**Figure 4:**
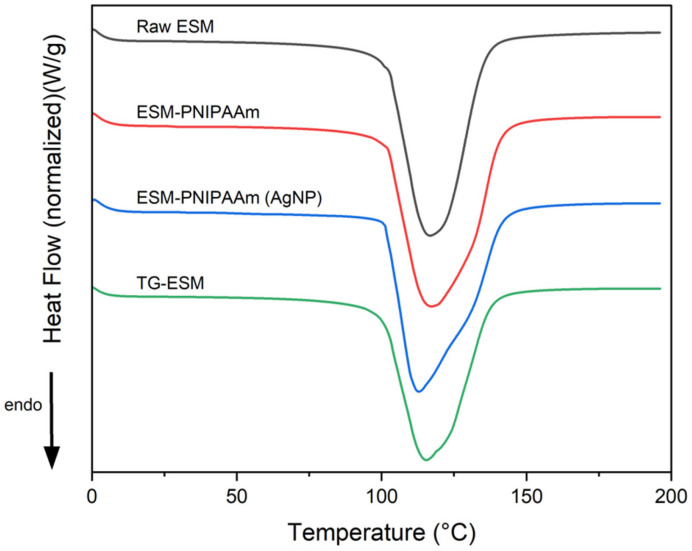
DSC thermogram of the raw and modified ESM. Distinct endothermic decomposition peak at approximately 160 °C, associated with the decomposition of collagen (Ramp 20 °C/min, 0 to 250 °C).

Thermal behaviour of the raw and modified membranes were assessed using a DSC-based protocol. The general thermograms and thermal profiles are summarised in Figure 4 and Table 1, respectively. All membrane samples show a distinct endothermic decomposition peak at ~115 °C. Similar endothermic peaks were reported by Torres et al. [24], associating this to the decomposition of collagen within the membrane, resulting from the rupture of hydrogen bonds and the rearrangement of the helical structure into random chain configuration. Table 1 shows that there is no significant difference between the % mass loss, onset temperature, peak temperature, or enthalpy for the raw and modified samples, indicating that such modifications do not alter the thermal properties of the membranes.

### 3.3. Mechanical Properties

**Figure 5:**
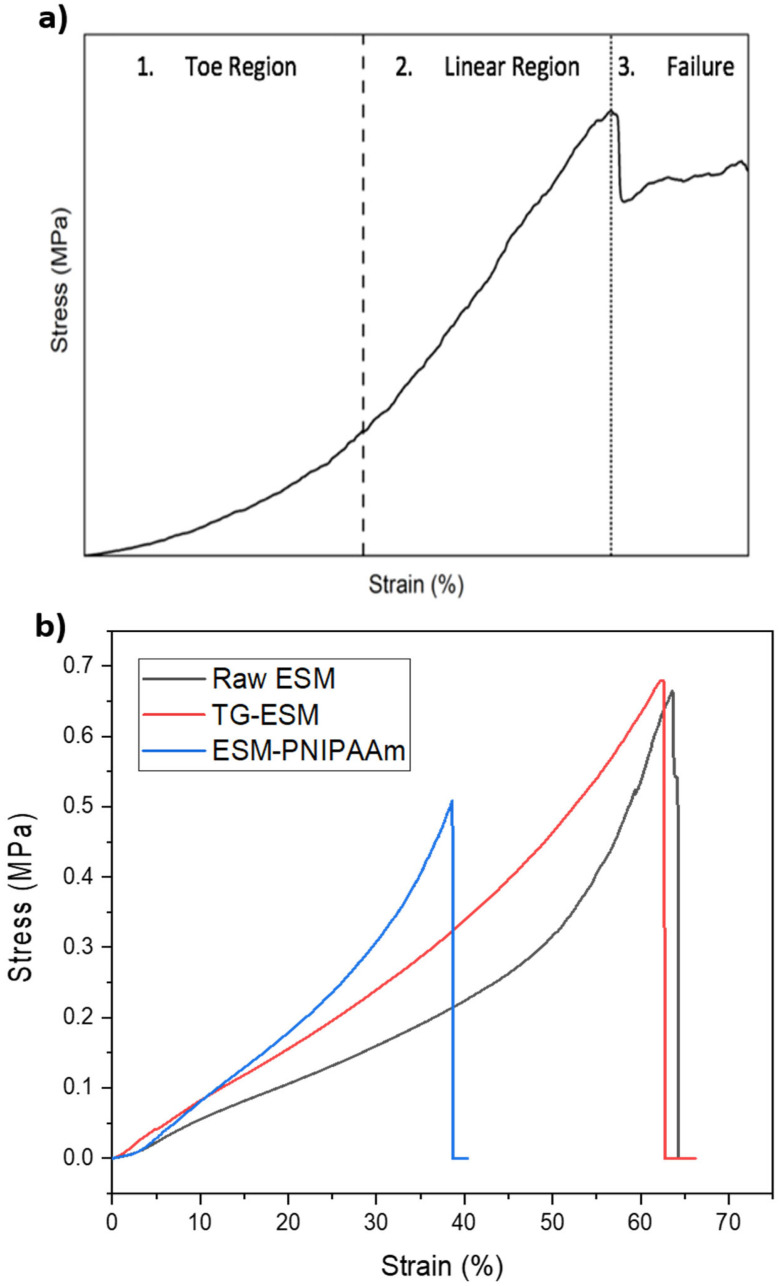
Tensile profile of the raw and modified ESM. (**a**) The general stress-strain profile of the ESM. (**b**) representative stress-strain curves of the raw and modified ESM (ESM-PNIPAAm (AgNP) and TG-ESM).

The tensile properties of the membranes were analysed from the stress-strain curves obtained from DMA profiling. Representative stress-strain curves of the raw and modified membranes are shown in Figure 5a,b. The membranes show a predominantly elastic behaviour (Figure 5b), in line with previous publications reporting the mechanical behaviour of collagen-based materials [28,29], the ESM shows linear and non-linear regions. Accordingly, within the non-linear (toe) region, a minimal amount of stress is required initially to deform the membrane as collagen fibres require alignment in the direction of the stress [30]. Within the second region, the membrane’s stiffness increases with elongation until the UTS is reached and the membrane ruptures (failure).

The mechanical measurements, including the Young’s modulus, UTS and % elongation at break, are summarised in Table 2. It can be noted from the data that significant differences between the % elongation at break of the raw ESM and ESM-PNIPAAm (*p* < 0.05) and raw ESM and TG-ESM (*p* < 0.005) exists. Similarly, a significant difference between the Young’s modulus of the raw ESM, TG-ESM (*p* < 0.005) and the raw ESM and ESM-PNIPAAm (*p* < 0.05) can be seen. A significant difference between the UTS of the raw ESM and TG-ESM (*p* < 0.005) was also observed. No significant difference was observed between the mechanical properties of the raw ESM and ESM-I. As such, it can be proposed such modifications increase the tensile strength of the membrane, due to alterations in the fibrillar structure of the material. The tensile properties indicate the membrane is a seemingly strong material and further modification, particularly crosslinking, improved these properties.

### 3.4. Drug Release Profile—ESM-PNIPAAm

**Figure 6:**
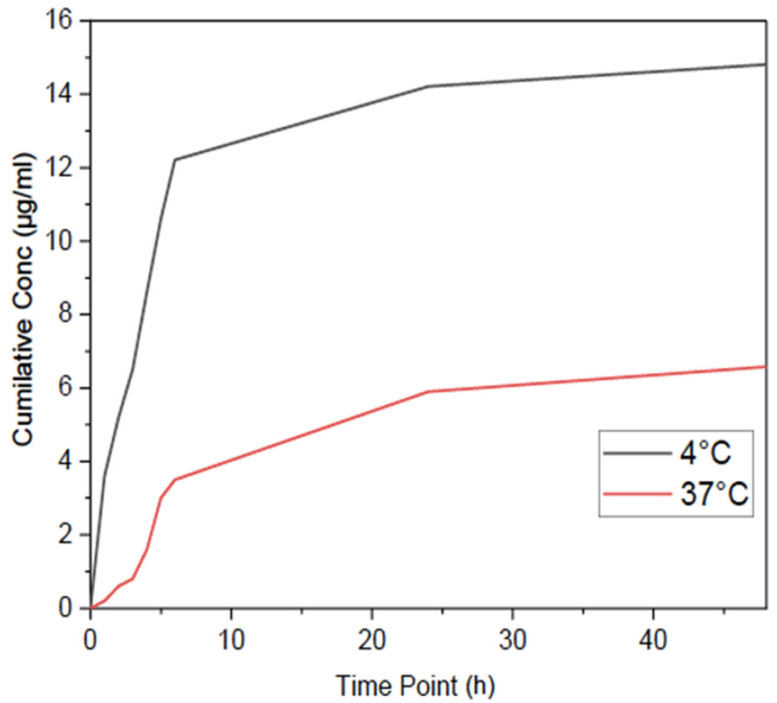
UV-Vis spectra of drug release from ESM-PNIPAAm (AgNP) at temperatures above (37° C) and below (4° C) the LCST. Samples were assessed at 380 nm, which corresponded to the absorbance wavelength of the commercially available AgNP (i.e., 380–405 nm). ESM-PNIPAAm (AgNP) samples at 4 °C show an initial burst release profile within the first 6 h, in which most of the NPs are released. Samples at 37 °C show little to no release of NP, relating to the thermoresponsive properties of PNIPAAm.

Ultraviolet-visible (UV-Vis) spectroscopy was used to monitor the release of AgNP from the ESM-PNIPAAm (AgNP)-modified membrane. Samples were assessed at 380 nm, which corresponded to the absorbance wavelength of the commercially available AgNP (i.e., 380–405 nm). To establish the thermoresponsive properties of PNIPAAm, the release of AgNP was monitored at 4 °C and 37 °C (Figure 6). A significant increase of AgNP was observed within the first 8 h from the ESM-PNIPAAm (AgNP) at 4 °C. The spectra indicate that at temperatures below the LCST (4 °C), the NP are entirely released within 1–8 h, after which the concentration plateaus.

### 3.5. In Vitro Cytotoxicity

**Figure 7:**
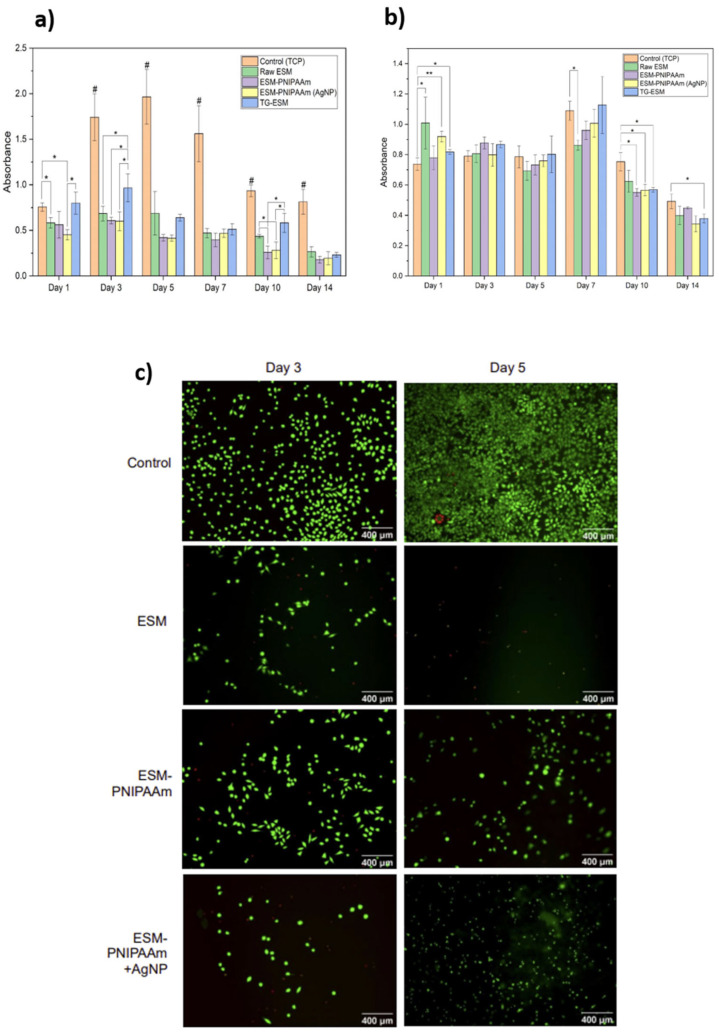
Human dermal fibroblasts (adult) (HDFa) cell viability, proliferation and adhesion assay. (**a**) Cell metabolic activity of HDFa cells cultured on TCP, raw and modified (ESM-PNIPAAm, ESM-PNIPAAm (AgNP), TG-ESM) membranes over fourteen days. Data are represented as mean ± SD (*n* = 4). (**b**) LDH release of HDFa cells cultured on TCP, raw and modified membranes (ESM-PNIPAAm, ESM-PNIPAAm (AgNP), TG-ESM) over fourteen days. Data are represented as mean ± SD (*n* = 4). (**c**) Cell viability and cytotoxicity was demonstrated by Live/DeadTM staining of cells seeded on the raw ESM, PNIPAAm adn ESM-PNIPAAm (AgNP) on days 3 and 5 under the fluorescence microscope. The stain causes live cells to fluoresce green and the dead cells to fluoresce red. Fluorescence imaging of cells seeded on ESM-PNIPAAm and ESM-PNIPAAm (AgNP) show a sustained cell viability after 3 and 5 days of incubation with the samples. In contrast, cells seeded on the raw ESM showed a decreased cell viability between 3 and 5 days. Sample is significantly different to all other samples within group with * *p* < 0.05, ** *p* < 0.005, and # *p* < 0.001).

Cell mitochondrial activity and cell death of the HDFa cells were assessed using the MTS and LDH assays, respectively, following culture on the raw and modified membrane samples for up to fourteen days (Figure 7a,b).

L929 cells were seeded on samples of the raw ESM, ESM-PNIPAAm and ESM-PNIPAAm (AgNP) and stained with a Live/DeadTM cytotoxicity assay kit to determine the in vitro viability and cytotoxicity of the dressing. The effect of the sample materials on cell viability was qualitatively validated using fluorescence microscopy (Figure 7c) in which the control groups (TCP) exhibited very high cell viability and contained minimal dead cells after 3 and 5 days, while after 3 days of incubation with each of the test samples, most cells remained viable, although the cell number was not as high as the control. Interestingly, after 5 days, the ESM-PNIPAAm and ESM-PNIPAAm (AgNP) samples showed a sustained cell viability.

## 4. Conclusions

An optimized protocol for isolating the ESM in its native state using an acid-based protocol has been outlined in this study. Physico-mechanical characterisation techniques were used to determine the ESM’s defined properties. Overall, the generated data suggests that the ESM can be employed in regenerative applications, particularly to improve regeneration following dermal injuries and trauma. Moreover, a thermoresponsive PNIPAAm polymer was successfully added to the membrane and addition AgNP subsequently loaded. The drug release profile of the fabricated dressing indicated a “burst” release of NP within the first eight hours at temperatures below its LCST (4 °C). In addition to having regenerative applications, the proposed material could also be used for drug delivery applications in the future when release and retention profiles are optimized.

Biocompatibility of the ESM-PNIPAAm (AgNP) dressing was also validated using cell culture, and a minimal adverse effect was observed along with sustained cell viability. Thus, these results suggest that the ESM, particularly when combined with the PNIPAAm hydrogel, is effective at improving wound healing and regeneration following dermal injury. It is even more relevant to use the ESM because it is a high-quality, readily available waste material.

## Figures and Tables

**Table 1:** Thermal properties of the raw and modified ESM. All values are expressed as mean ± SD for *n* = 5 (*p* < 0.05).

Sample Type	% Mass Loss	Onset Temp (°C)	Peak Temp (°C)	Enthalpy (J/g)
Raw ESM	73.607 ± 7.624	100.67 ± 0.707	113.817 ± 2.586	1838.167 ± 145.858
ESM-PNIPAAm	62.724 ± 8.874	99.544 ± 1.221	114.122 ± 2.102	1750.52 ± 176.214
ESM-PNIPAAm (AgNP)	67.9 ± 1.718	101.41 ± 2	111.493 ± 1.44	2041.933 ± 72.403
TG-ESM	73.64 ± 6.399	100.592 ± 1.253	112.2 ± 2.032	1745.067 ± 342.617

**Table 2:** Mechanical profile of the raw and modified membrane. All values expressed as mean ± SD, *n* = 5 (* *p* < 0.05, ** *p* < 0.005).

Sample Type	% Elongation	UTS (MPa)	Young’s Modulus (MPa)
Raw ESM	15.248 ± 2.819	0.419 ± 0.035	2.242 ± 0.309
ESM-PNIPAAm	27.045 ± 8.352 (*)	0.348 ± 0.124	2.854 ± 0.192 (*)
TG-ESM	37.678 ± 1.739 (**)	0.612 ± 0.051 (**)	3.892 ± 0.190 (**)

## Data Availability

Not applicable.
